# Quality assessment of different marketed brands of *Dasamoolaristam*, an Ayurvedic formulation

**DOI:** 10.4103/0974-7788.59937

**Published:** 2010

**Authors:** V. Kalaiselvan, Ankur Kalpeshkumar Shah, Falgun Babulal Patel, Chainesh Narendrabhai Shah, M. Kalaivani, A. Rajasekaran

**Affiliations:** Department of Pharmacognosy, KMCH College of Pharmacy, Kovai Estate, Kalapatti Road, Coimbatore, 641035, India; 1 Department of Indian Pharmacopoeia Commission, Sector 23, Raj Nagar, Ghaziabad, UP-201 02, India

**Keywords:** *Dasamoolaristam*, alcohol content, Microbial load, extractive value, *E. coli*

## Abstract

*Arista* is a classical Ayurvedic preparation that is typically used as a digestive and cardiotonic. The present Investigation evaluated five different brands of *Dasamoolaristam* available in the market as per WHO and Indian Pharmacopoeial specifications. Various physicochemical parameters such as alcohol-soluble extractive, water-soluble extractive, total ash, acid-insoluble ash, total solid, and alcohol content were determined. The present investigation reveals that all the preparations contain acceptable levels of alcohol (less than 12% v/v). However, the preparations were found to contain unacceptable limits of microbial load although all showed the absence of *Escherichia coli*, *Salmonella* species*,* and *Staphylococcus aureus*.

## INTRODUCTION

*Ayurveda* comprises of various types of medicines including the fermented forms, namely, aristas (fermented decoctions) and *asavas* (fermented infusions). In Ayurvedic aristas, *Dasamoolaristam* is a unique combination of ten herbs, where ‘*dasa’* means ten and ‘*moola’* means root. These preparations are regarded as valuable therapeutic formulations due to their efficacy and desirable features. They are generally prepared by soaking the drug, either in powdered form or in the form of decoction (*Kasaya*), in a solution of sugar or jaggery, for a specified period of time. During this period, the preparation undergoes a process of fermentation to generate alcohol, thus facilitating the extraction of the active principles contained in the drugs.[1] The bulk of knowledge on these fermented decoctions, however, remains lacking in documentation, validation, and determination of marker compounds.

The quality assessment of herbal formulations is of paramount importance in order to justify their acceptability in modern systems of medicine. One of the major problems faced by the herbal drug industry is the unavailability of rigid quality control profiles for herbal materials and their formulations. Regulatory bodies have laid down the standardization procedures and specifications for ayurvedic preparations. In India, the Department of AYUSH, Government of India, launched a Central Scheme to develop a standard operating procedure for the manufacturing process to develop pharmacopoeial standards for Ayurvedic preparations.

Various scientists have actively participated to validate Ayurvedic formulations.[2–4] It is mandatory to carry out the microbiological limit test to ensure whether the product is free from risk. Herbal formulations available in the market are usually not properly standardized and are not assessed for their quality. As the use of herbal formulations by patients is increasing, there is an urgent need for pharmacists and physicians to have knowledge about the safety and efficacy of these preparations.

## MATERIALS AND METHODS

Five different brands of *Dasamoolaristam* were purchased from the Kottakkal Arya Vaidya Pharmacy, Coimbatore. All the samples were stored in the refrigerator at 8°C and collected for experiments under aseptic conditions.

### Physical and Physiochemical evaluations

All physical evaluations such as alcohol content, total solid content, water-, and alcohol-soluble extractive values were determined as per the method prescribed in the Indian Pharmacopoeia.[5]

### Microbial evaluations

#### Test for contaminating fungus (yeast and mould)

Ten grams of the sample were suspended in 100 mL of phosphate buffer, pH 7.2. One milliliter of the preparation was added to 15 mL of liquefied potato dextrose agar medium in two petridishes and incubated at 25°C for seven days. The dishes were then observed and the numbers of colonies counted.[5]

### Total aerobic microbial count

Ten grams of the sample were suspended in 100 mL of buffered sodium chloride-peptone solution, pH 7. Subsequently, 0.1% w/v of polysorbate 80 was added to assist the suspension of poorly wettable substances. One milliliter of the preparation and about 15 mL of the liquefied casein soyabean digest agar were added to two petridishes kept at not more than 45°C and incubated at 30 to 35°C for four days. The dishes were observed and the numbers of colonies counted.

### Test for *Escherichia coli*

Ten grams of the sample were suspended in 100 mL of buffered lactose broth by shaking in a sterile screw-capped jar. Subsequently, 0.1% w/v of polysorbate 80 was added to assist the suspension of poorly wettable substances. One milliliter of the preparation was transferred into a sterile screw-capped container and 50 mL of nutrient broth added. The mixture was then shaken and allowed to stand for 1 h and then shaken again. The cap was loosened and the jar incubated at 37°C for 24 h. The dishes were tested for the presence of acid and gas as per the standard procedure.

### Test for *Salmonella*

One gram of the sample was suspended with 100 mL of nutrient broth in a sterile screw-capped jar, allowed to stand for 4 h and shaken. The cap was loosened and the jar incubated at 35 to 37°C for 24 h. One milliliter of the enrichment culture was added to each of two tubes containing 10 mL of selenite F broth and tetrathionate bile-brilliant green broth separately, and incubated at 36 to 38°C for 48 h. Each of these two cultures were subcultured onto bismuth sulphite agar and brilliant green agar. The plates were incubated at 36 to 38°C for 18 to 24 h and observed for the presence of black-green or pink colonies respectively.

### Test for *Staphylococcus aureus*

Ten grams of the sample were suspended by shaking with 100 mL of nutrient broth in a sterile screw-capped jar and allowed to stand for 4 h and then shaken again. The cap was loosened and the jar was incubated at 35 to 37°C for 24 h. One milliliter of the enrichment culture was added to soyabean-casein digest medium which was examined for the presence of growth. A portion of medium was streaked on the surface of Vogel-Johnson agar and Mannitol-salt agar medium. Plates were then incubated at 36 to 38°C for 18 to 24 h and observed for the presence of black and yellow colonies surrounded by yellow zones.

## RESULTS

Physical and physiochemical parameters such as pH, total solid content, water-and alcohol-soluble extractive, and alcohol content were determined using standard pharmacopoeial methods. Alcohol percentage (v/v) in each of the tested brands of *Dasamoolaristam* was found to be different. The maximum alcohol content (12%) was found in brand 2 and the minimum (4%) was found in brands 1 and 4. The remaining brands 3 and 5 had alcohol contents of 6 and 7% respectively [Table 1]. The results thus highlighted that the levels of alcohol in *Dasamoolaristam* were lower than those in fortified wines and distilled spirits. The total alcohol content for all the brands was under the acceptable limit.

**Table 1:** Physical and Physiochemical evaluation of different brands of *Dasamoolaristam*

Name of the test	Brand _1_	Brand _2_	Brand _3_	Brand _4_	Brand _5_
pH	3	2.5	3	2.5	2.5
Total solid content (% w/v)	21.3	22.1	19.4	3021	
Alcohol content (%v/v)	4	11	6	4	7
Water-soluble extractive (% w/w)	10.2	11.5	9.5	10.8	10.3
Alcohol-soluble extractive (% w/w)	10.7	9.5	11.2	10.5	11.3

**Figure 1:**
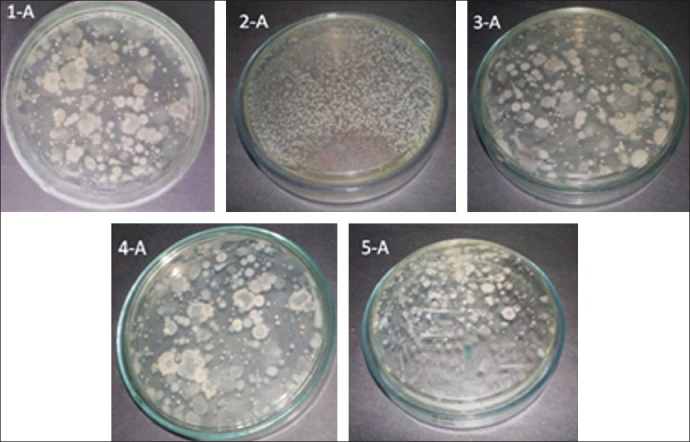
Evaluation of total aerobic load in different brands of *Dasamoolaristam*

**Figure 2:**
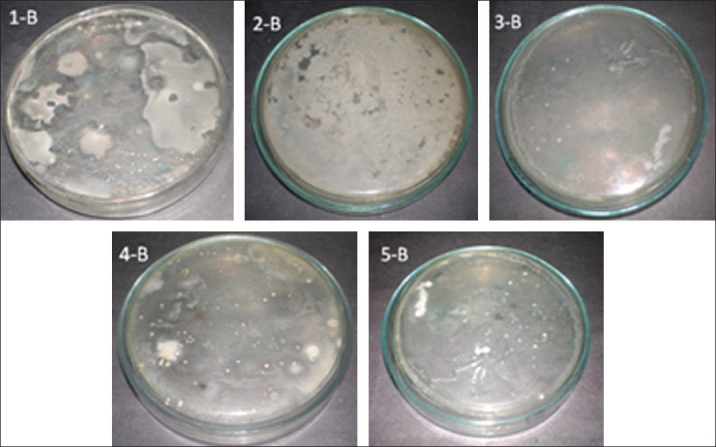
Evaluation of total fungal load in different brands of *Dasamoolaristam*

The total aerobic counts of brands 1, 3, 4, and 5 were found to be 1.9×10^3^, 2.3×10^3^, 1.7×10^3^ and 2.3×10^3^ CFU/ mL respectively. The growth Was unable uncountable in brand 2 [Table 2 and Figure 1]. Fungal counts in brands 1, 3, 4 and 5 were found to be 1.7×10^3^, 2.1×10^3^, 1.6×10^3^ and 2.4×10^3^ CFU/mL; again, the growth was unable uncountable in brand 2 [Table 2 and Figure 2]. All the preparations were found to be negative for the presence of *Escherichia coli*, *Salmonella* species, and *Staphylococcus aureus*.

**Table 2:** Microbiological evaluation of different marketed brands of *Dasamoolaristam*

Microbial test	Brand _1_	Brand _2_	Brand _3_	Brand _4_	Brand _5_
	
			CFU/mL)		
Fungus	1.7×10^3^	NC	2.1×10^3^	1.6×10^3^	2.4×10^3^
Total aerobic count	1.9×10^3^	NC	2.3×10^3^	1.7×10^3^	2.3×10^3^
*Escherichia coli*	-	-	-	-	-
*Salmonella* spp	-	-	-	-	-
*Staphylococcus aureus*	-	-	-	-	-

NC - Not countable; pharmacopoeia limits for total aerobic and fungal count not more than 300 and 100 CFU/mL respectively

## DISCUSSION

India has a great diversity in medicinal herbal resources. More than 70% of the Indian population uses herbal drugs for the treatment of various diseases, and the manufacture of these medicines is mushrooming. Traditional herbal medicines and their preparations have been widely used in India as well as abroad for many years.[6] But there are only few industrial organizations in India who carry out quality assessment on herbal drugs. In spite of standardization parameters and quality assessment, the share of India in the global herbal market is not up to the mark.[7] In traditional systems of medicine, the drugs are primarily dispensed as water decoctions or ethanolic extracts, fresh plant parts, juices, or crude powder. Therefore, medicinal plant parts should be authentic and free from microbial contamination. This is the reason why the World Health Organization has set specific guidelines for the assessment of the safety, efficacy, and quality of herbal medicines as a prerequisite for global harmonization.[8] Still, very few Ayurvedic industries follows Good manufacturing practices (GMP) and are ISO-certified.

Microbial and fungal contamination not only affects the chemical composition but also decreases the therapeutic potency of herbal drugs.[4] Microbial contamination of herbal drugs is a major impediment that prevents India from becoming an herbal giant. Therefore, fungal contamination of drugs, especially raw materials, should be prevented during the manufacture of these preparations. Plant materials used for medical purposes should be properly stored and the growth of bacteria and fungi should be inhibited.[9] India can be a major player in the global herbal market if herbal preparations are manufactured according to regulatory guidelines.

In the present investigation, we have assessed the quality of different brands of the marketed Ayurvedic preparation, *Dasamoolaristam*. The preparations were purchased from the Kottakkal Arya Vaidya Pharmacy, Coimbatore, and stored in a refrigerator before conducting the microbiological limit test under aseptic conditions. The present findings have shown that the alcohol content of all brands of *Dasamoolaristam* are lower than the acceptable limit of alcohol content. As compared to Pharmacopoeial specifications. Unfortunately, all the five different brands of *Dasamoolaristam* were found to contain bacterial and fungal loads above the acceptable limits.

## CONCLUSIONS

The present investigation found microbial loads that were higher than the acceptable limits in some leading brands of *Dasamoolaristam*. Regulatory bodies and the government sector should implement stringent policies to regulate and monitor the manufacture and marketing of herbal formulations.
